# Characterization of the complete chloroplast genome of *Ajuga forrestii* (Lamiaceae), a medicinal plant in southwest of China

**DOI:** 10.1080/23802359.2019.1689193

**Published:** 2019-12-05

**Authors:** Ai-En Tao, Fei-Ya Zhao, Cong-Long Xia

**Affiliations:** a College of Pharmacy and Chemistry, Dali University, Dali, China;; b School of Medicine, Tourism and Culture College of Yunnan University, Lijiang, China;; c Key Laboratory, Yunnan Provincial Higher Education Institutions for Development of Yunnan Daodi Medicinal Materials Resources, Yunnan, China

**Keywords:** *Ajuga forrestii*, chloroplast, Illumina sequencing, phylogeny

## Abstract

*Ajuga forrestii* is a medicinal plant commonly used in southwest of China. In this study, we sequenced the complete chloroplast (cp) genome sequence of *A. forrestii* to investigate its phylogenetic relationship in the family Lamiaceae. The chloroplast genome of *A. forrestii* was 150,492 bp in length with 38.3% overall GC content, including a large single copy (LSC) region of 82,148 bp, a small single copy (SSC) region of 17,160 bp and a pair of inverted repeats (IRs) of 25,592 bp. The cp genome contained 112 genes, including 79 protein coding genes, 29 tRNA genes, and 4 rRNA genes. The phylogenetic analysis indicated *Ajuga* was closely related to *Scutellaria*.

*Ajuga* is a great genus of the Lamiaceae family, which includes 300 species in the world. Most of them are widely distributed in Europe, Asia, Africa, Australia and North America (Israili and Lyoussi 2009; Cocquyt et al. 2011). There are 18 species in China (Li and Ian 1994). Plants of this genus have been widely used in traditional Chinese medicine for thousands of years (Jiangsu New Medical College 1977). Among these species, *Ajuga forrestii* is widely distributed in southwest China which have been used in local medicine as anti-inflammatory, antioxidant, cytotoxic, analgesic, or antibacterial activity (Toiu et al. 2018). However, up to now for such medicinal plant, many studies have mainly focused on describing its chemical compositions (Wang et al. 1994; Xiong et al. 2013; Chen et al. 2018), with little involvement in its molecular biology, so that no comprehensive genomic resource is conducted for it. Here, we report the chloroplast genome sequence of *A. forrestii* and find its internal relationships within the family Lamiaceae.

Fresh and clean leave materials of *A. forrestii* were collected from Yulong county, Yunnan, China (N27°00′51″, E100°14′45.7″), and the plant materials and a voucher specimen (No. TC19) were Tourism and Culture College of Yunnan University (Lijiang). Total genomic DNA was extracted using the improved CTAB method (Doyle 1987; Yang et al. 2014), and sequenced with Illumina Hiseq 2500 (Novogene, Tianjin, China) platform with pair-end (2 × 300 bp) library. The raw data was filtered using Trimmomatic v.0.32 with default settings (Bolger et al. 2014). Then paired-end reads of clean data were assembled into circular contigs using GetOrganelle.py (Jin et al. 2018) with *Ajuga reptans* (No. KF709391) as reference. Finally, the cpDNA was annotated by the Dual Organellar Genome Annotator (DOGMA; http://dogma.ccbb.utexas.edu/) (Wyman et al. 2004) and tRNAscan-SE (Lowe & Chan 2016) with manual adjustment using Geneious v. 7.1.3 (Kearse et al. 2012).

The circular genome map was generated with OGDRAW v.1.3.1 (Greiner et al. 2019). Then the annotated chloroplast genome was submitted to the GenBank under the accession number MN518848. The total length of the chloroplast genome was 150,492 bp, with 38.3% overall GC content. With typical quadripartite structure, a pair of IRs (inverted repeats) of 25,592 bp was separated by a small single copy (SSC) region of 17,160 bp and a large single copy (LSC) region of 82,148 bp. The cp genome contained 112 genes, including 79 protein coding genes, 29 tRNA genes, and 4 rRNA genes. Of these, 17 genes were duplicated in the inverted repeat regions, 10 genes, and 6 tRNA genes contain one intron, while two genes (*ycf3* and *clpP*) have two introns.

To investigate its taxonomic status, a total of 24 cp genome sequences of Lamiaceae species were downloaded from the NCBI database used for phylogenetic analysis. After using MAFFT V.7.149 for aligning (Katoh and Standley 2013), a neighbor-joining (NJ) tree was constructed in MEGA v.7.0.26 (Kumar et al. 2016) with 1000 bootstrap replicates and four Solanaceae species (*Nicotiana otophora*: NC_032724, *Solanum melongena*: MF818319, *Physalis peruviana*: MH019242, and *Capsicum chinense*: NC_030543) were used as outgroups. The results showed that *Ajuga* was closely related to *Scutellaria* (Figure 1). Meanwhile, the phylogenetic relationship in Lamiaceae was consistent with previous studies and this will be useful data for developing markers for further studies.

**Figure 1.**
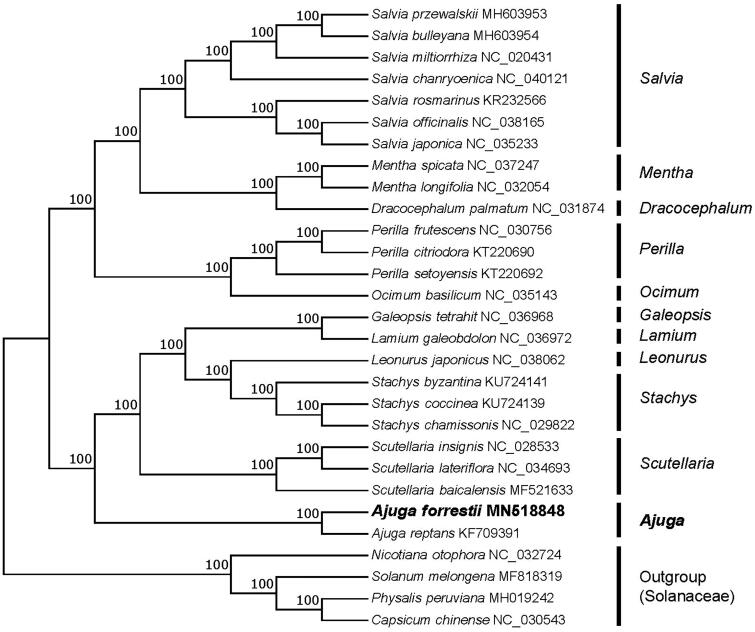
Neighbor-joining (NJ) tree of 25 species within the family Lamiaceae based on the plastomes using four Solanaceae species as outgroups.
